# On the mechanism of painful burn sensation in tattoos on magnetic resonance imaging (MRI). Magnetic substances in tattoo inks used for permanent makeup (PMU) identified: Magnetite, goethite, and hematite

**DOI:** 10.1111/srt.13281

**Published:** 2023-03-16

**Authors:** Jørgen Serup, Kasper Køhler Alsing, Ole Olsen, Christian Bender Koch, Rasmus Hvass Hansen

**Affiliations:** ^1^ The Tattoo Clinic, Department of Dermatology Copenhagen University Hospital, Bispebjerg Copenhagen Denmark; ^2^ Medico Chemical Lab Vedbæk Denmark; ^3^ Department of Chemistry Copenhagen University Copenhagen Denmark; ^4^ Section for Radiation Therapy, Department of Oncology, Center for Cancer and Organ Diseases Copenhagen University Hospital, Rigshospitalet Copenhagen Denmark

**Keywords:** biomagnetism, cosmetic tattoo, iron oxide, magnetic field, magnetism, neurosensitive reaction, ore minerals, tattoo burn, tattoo complication, tattoo pain

## Abstract

**Background:**

Persons with cosmetic tattoos occasionally experience severe pain and burning sensation on magnetic resonance imaging (MRI).

**Objective:**

To explore the culprit magnetic substances in commonly used permanent makeup inks.

**Material and methods:**

20 inks used for cosmetic tattooing of eyebrows, eyeliners, and lips were selected. Ink bottles were tested for magnetic behavior with a neodymium magnet. Eight iron oxide inks qualified for the final study. Metals were analyzed by Inductively Coupled Plasma Mass Spectrometry (ICP‐MS). The magnetic fraction of inks was isolated and analyzed by X‐ray fluorescence (XRF). Magnetic iron compounds were characterized by Mössbauer spectroscopy and powder X‐ray diffraction (XRD).

**Results:**

ICP‐MS showed iron in all magnetic samples, and some nickel and chromium. Mössbauer spectroscopy and XRD detected ferromagnetic minerals, particularly magnetite, followed by goethite and hematite.

**Conclusion:**

This original study of cosmetic ink stock products made with iron oxide pigments reports magnetic impurities in inks for cosmetic tattooing, e.g., magnetite, goethite, and hematite. These may be the main cause of MRI burn sensation in cosmetic tattoos. The mechanism behind sensations is hypothesized to be induction of electrical stimuli of axons from periaxonal pigment/impurity activated by magnetic force. Magnetite is considered the lead culprit.

## 1 INTRODUCTION

Hazards and adverse effects from ferromagnetic material such as metal implants may occur in MRI procedures.^1^, ^2^ Tattoo ink stock products are known to contain metallic ingredients and impurities;^3^, ^4^, ^5^ thus, adverse reactions may occur in tattooed skin on exposure to forceful magnetic fields.^6^ A review of the literature finds reports of burn sensations of varying degree without associated physical signs albeit erythema and edema in the tattoo occasionally may occur. The literature has misinterpreted the feeling of burn and claimed the sensation to be a real thermal insult induced by MRI;^7^, ^8^, ^9^, ^10^ inks exposed to MRI show no temperature increase.^7^ Permanent makeup is in the medical literature indicated to carry a special risk of MRI‐induced complication due to the commonly used iron oxide pigments in this type of inks.^6^, ^7^ MRI adverse events of decorative tattoos on trunk and extremities have been sporadically reported, but events are exceptional relative to the popularity of these adornments.^11^, ^12^

Iron oxide pigments of high purity are usually not magnetic, and MRI events of tattoos made with this class of chemicals are suspected to be due to magnetic contaminants or impurities in the crude raw material used in production. Ink production is industrial, widely unregulated, and not quality assured through good manufacturing practice (GMP) standards.

The word magnet comes from the ancient Greek city Magnesia founded by the Magnete tribe; rocks in this city are ferromagnetic. Iron oxide pigments in tattoo inks often originate from some natural source of raw material supply and, thus, are likely to be variably contaminated with metals and minerals, including nickel, cobalt, chromium, and copper.^3^, ^5^, ^7^, ^13^ However, even high iron oxide content is not automatically associated with magnetism, as the magnetic properties depend on several chemical and physical factors such as oxidation level, the configuration of electrons in the material's atoms, and the crystal structure. Thus, some iron oxides may have paramagnetic or ferromagnetic properties; meaning it is attracted by magnets. Some can in the laboratory become permanently magnetic.

The aim of this study was to identify magnetic substances in commercial ink stock products often used in cosmetic tattooing. This was to our best knowledge not explored in the past. Identification of magnetic culprit substances in tattoo inks may result in the future manufacturing of MRI‐safe tattoo inks. Burn sensation in a tattoo often interrupts and invalidates the MRI procedure, and MRI is essential in the diagnosis of, for example, cancer.

## 2 MATERIAL AND METHODS

### 2.1 Selection of commercial ink stock products by magnets test: initial off‐the‐shelf test

One investigator (J. Serup) visited a local cosmetic tattoo studio, Diana Hvas Perfecting Beauty, Rødovre, Denmark. Ink stock products exclusively made for cosmetic tattooing e.g., eyebrows, eyeliners, and lips were randomly selected from the studio ink portfolio. A simple test using a handheld magnet was introduced to identify ink bottles with magnetic behavior.^14^ In this primary screening, using a solid neodymium magnet strength of 0.3 tesla (T) measured with a handheld Gaussmeter, the ink bottles were exposed to a static magnetic field. A product was deemed test positive if the whole ink bottle when lying horizontally could be rolled on a flat surface, drawn by the magnet. The test is used with caution as asymmetry of the ink bottle, labeling, and sometimes a metal nut for stirring might produce false‐positive or ‐negative outcomes. Therefore, in case of doubtful outcomes, a confirmatory test was conducted. In a transparent Petri dish, a single drop of ink was dosed in the center of the dish. A handheld neodymium magnet was moved under the Petri dish, and the ink drop was visually inspected for surface structure changes referenced to a Rosensweig instability, or horizontal move of the entire drop of ink following movement of the magnet under the bottom of the Petri dish.^15^

**FIGURE 1:**
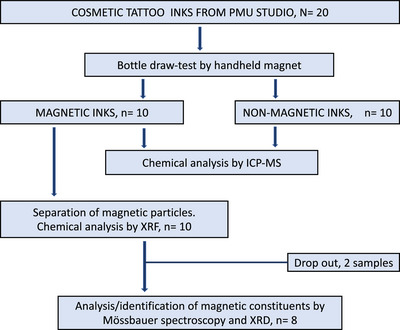
Flowchart of the study

All ink bottles were categorized as magnetic or nonmagnetic (Figure 1). Nonmagnetic samples were with one exception excluded from more detailed analysis. 6 M was reconsidered and concluded nonmagnetic and served as a negative control in further testing.

### 2.2 Chemical analysis of cosmetic tattoo products showing magnetic behavior in the initial off‐the‐shelf test

Enrolled inks were analyzed by Inductively Coupled Plasma Mass Spectrometry (ICP‐MS) after microwave dissolution in nitric acid and hydrochloric acid with measurement of total concentrations (sum of soluble and solid contents) of cadmium, chromium, copper, nickel, lead, zinc, iron, and mercury. Chemical analyses were performed by Medico Chemical Lab, Vedbæk, Denmark.

### 2.3 Separation of magnetic particles in magnetic inks

In a 100 ml beaker, 1.0 gram of each magnetic tattoo ink was weighed. The ink was then dissolved in 25 mL of demineralized water. To isolate the magnetic pigment particles, a powerful permanent magnet (diameter 2.4 cm × height 1.5 cm) was held under the beaker, and the ink dilutions were centrifuged in the glass for 30 s. This was to locate and retain the ferromagnetic particles after the supernatant was gently discarded. The procedure was repeated until suspensions of particles were transparent; 40–60 extractions were required.

The isolated magnetic particles were then mixed with a small amount of demineralized water in a pre‐weighed vial and placed on a heating plate for 8 h to dehydrate. Thereafter, the dehydrated pigment powder was weighed and analyzed by solid‐state methodology (see Section 2.4).

### 2.4 Analysis of elements in separated magnetic particles by X‐ray fluorescence (XRF)

A Niton™ XL3t GOLDD, a handheld high‐performance portable X‐ray fluorescence elemental analyzer from Thermo Scientific was used.^16^

### 2.5 Mössbauer spectroscopy to identify magnetic chemicals/constituents

Fe‐57 Mössbauer spectra were obtained at room temperature (298 K) on air‐dry powdered sample packed into a Perspex sample holder, using a conventional constant acceleration spectrometer and a collimated source of Co‐57 in Rh. The spectrometer was calibrated using a 12.5 mm foil of natural Fe at room temperature, and isomer shifts were given with respect to the center of the spectrum of the absorber. In this experiment, no thickness corrections were applied to the spectra. Spectra were fitted using a combination of doublet and sextet components having Lorentzian line shapes.

### 2.6 X‐ray powder diffraction

Powder X‐ray diffraction (PXRD) analyses were conducted with a Bruker D8 Advance diffractometer using CuKα radiation. The CuKβ radiation was suppressed using a Ni filter. All analyses were performed at room temperature in the angular range 10–60° 2θ with a step size of 0.02° 2θ and a dwell time of 10 s per step. The energy window of the Lynxeye detector was set to reduce the noise caused by the Fe fluorescence effects excited by the Cu emission radiation from the iron‐rich samples.

## 3 RESULTS

Twenty random cosmetic ink products from the cosmetic tattoo clinic were enrolled (flowchart; Figure 1). Tables 1A and 1B show the ink stock products studied, and product label information on manufacturer, country of origin of the product, pigments by Color Index (CI) number according to label.

**TABLE 1A:** Cosmetic tattoo ink stock products tested magnetic with a handheld magnet applied to bottle

	Brand	Name	Color	Color index (CI) number	Origin
1 M	NA	NA	Light Brown	NA	NA
2 M	Biopigments	Coco	Dark Brown	*“Organic and inorganic colorant”*	Poland
3 M	LI Pigments	Classic Brown	Brown	77499, 77491, 77402	US
4 M	DOREME	231 Espresso	Brown	77499, 77491, 77492, 77891	Korea
5 M	Meditouch	Khaki Brown	Light Brown	*“May contain iron oxides/titanium dioxide”*	Korea
6 M^a^	PermaBlend	Burnt Sienna	Nutty Brown	77891, 21095, 12477, 77266	US
7 M	DOREME Pigments	818 Olive	Olive green	NA	Korea
8 M	Bella	16 Brown	Brown	“Iron oxide”	Taiwan
9 M	Biotek	Light brown 1	Light brown	77891 77499, 77491, 77492, 77288	Italy
10 M	Purebeau	Monroe AB08	Brown	NA	Germany

^a^ False positive, repositioned and used in further analyses as a negative control.

NA, pigment not indicated on label.

**TABLE 1B:** Cosmetic tattoo ink stock products tested nonmagnetic with a handheld magnet

	Brand	Name	Color	Color index (CI) number	Origin
1C	A. Sivak	Tea rose	Light Brown	77891, 21119, 12477	Russia
2C	LIPigments	UN‐gray	Brown	*“*Contain iron oxides*” 77499, 77491, 77492*	US
3C	DOREME Pigments	41 Yellow	Yellow‐brown	“Iron oxide, titanium dioxide”	Korea
4C	Swiss Color	IB 32	Brown	200310, 561170, 77266	Switzerland
	International	medium brown			
5C	Mei Cha Brand	Watermelon Lip Candy	Red	*77891, 12475, 11781*	Korea
6C	Phi Brows Academy	Pink	Light red	73907, 56110, 77891	Germany
7C	Swiss Color International	MB 802‐2 Flamingo	Orange reddish	11767, 561170, 56110, 73907	Switzerland
8C	DOREME	709 Cherry Blossom	Red	77891, 56110, 56300, 77266	Korea
9C	Premier Pigments	Magenta Mauve	Purple‐red	“Organic and inorganic pigments”	US
10C	Cell‐line Embo Pigments	Brown	Brown	“Iron oxide, titanium oxide”	Korea

*N* = 10.

Ten inks were positive in the initial magnet test, with visual movement of the bottle drawn by the handheld magnet. Magnetic ink samples (1 M–10 M) are listed in Table 1A, and nonmagnetic samples in Table 1B. However, using the confirmatory Petri dish test, one sample (no. 6 M) turned out false positive. This was confirmed by no detection of iron or other metals in the chemical analysis program (Table 2). Sample 6 M passed the full analytical package and thus served as a negative control.

**TABLE 2:** Elements analyzed by ICP‐MS of magnetic inks

	The concentration of elements in ink stock products (mg/kg)							
	Cd	Cr	Cu	Ni	Pb	Zn	Hg	Fe
1 M	I.D.	570	I.D.	I.D.	I.D.	I.D.	I.D.	13 000
2 M	I.D.	42	7	22	9.6	870	I.D.	230 000
3 M	I.D.	I.D.	I.D.	I.D.	I.D.	I.D.	I.D.	24 000
4 M	I.D.	5	I.D.	I.D.	I.D.	I.D.	I.D.	78 000
5 M	I.D.	I.D.	I.D.	I.D.	I.D.	I.D.	I.D.	31 000
6 M^a^	I.D.	I.D.	I.D.	I.D.	I.D.	I.D.	I.D.	I.D.
7 M	I.D.	6.9	I.D.	3.7	I.D.	I.D.	I.D.	160 000
8 M	I.D.	19	40	17	I.D.	I.D.	I.D.	45 000
9 M	I.D.	5.6	I.D.	11	I.D.	64	I.D.	240 000
10 M	I.D.	I.D.	I.D.	I.D.	I.D.	I.D.	I.D.	25 000
1C	I.D.	I.D.	I.D.	I.D.	I.D.	I.D.	I.D.	I.D.
2C	I.D.	I.D.	I.D.	I.D.	I.D.	I.D.	I.D.	22 000
3C	I.D.	I.D.	I.D.	I.D.	I.D.	I.D.	I.D.	21 000
4C	I.D.	I.D.	I.D.	I.D.	I.D.	I.D.	I.D.	I.D.
5C	I.D.	I.D.	I.D.	I.D.	I.D.	I.D.	I.D.	I.D.
6C	I.D.	I.D.	I.D.	I.D.	I.D.	I.D.	I.D.	I.D.
7C	I.D.	I.D.	I.D.	I.D.	I.D.	I.D.	I.D.	60
8C	I.D.	I.D.	I.D.	I.D.	I.D.	I.D.	I.D.	I.D.
9C	I.D.	I.D.	I.D.	I.D.	I.D.	I.D.	I.D.	68
10C	I.D.	I.D.	I.D.	I.D.	I.D.	I.D.	I.D.	63

*Magnetic inks (1‐10M), N* = 9; nonmagnetic inks (1‐10C and 6M), *N* = 10+1.

^a^ 6 M nonmagnetic negative control.

Detection limit for individual metals: cadmium (CD): 0.05 mg/kg, chromium (Cr): 5 mg/kg, copper (Cu): 5 mg/kg, nickel (Ni): 3 mg/kg, lead (Pb): 4 mg/kg, zinc (Zn): 50 mg/kg, mercury (Hg): 0.1 mg/kg.

I.D., not detected above the detection limit.

Ten nonmagnetic cosmetic inks were used as controls (no. 1C–10C) in the first part of the analytical program, e.g., measurement of elements (Table 2). They were negative in both magnet tests; thus, controls were qualified as being nonmagnetic. Some nonmagnetic and all magnetic inks were declared to contain iron oxides according to the manufacturer's product label stating CI‐numbers CI77491, CI77492, and CI77499. All magnetic samples were exclusively made for eyebrows and eyeliner tattooing, whereas 50% (5/10) of the nonmagnetic samples were made exclusively for lip tattooing. These were made with organic pigments.

ICP‐MS chemical analysis of the magnetic samples 1 M–10 M showed a significant content of elements, predominantly iron (range 13.000–240.000 mg/kg), but also other elements: nickel, chromium, copper, zinc, and lead. No inks had traces of cadmium or mercury. Half of the nonmagnetic samples had minor traces of iron; no other metals were detected (Table 2).

After separation of the magnetic particles (1 M–10 M), the retained dry pigment powder presented with dark brown or black colors, thus, the same color as the ink stock product.

Analysis of the dry powder pigment with a handheld XRF device showed high proportions of iron expressed as a percentage, and minor proportions of titanium, chromium, magnesium, zinc, silicon, and sulfur (Table 3). Consistent finding of silicon indicates some ore is the origin of the raw material.

**TABLE 3:** Elements in dry powder sediment with concentrated magnetic pigment measured by handheld X‐ray fluorescence (XRD)

	Content of elements in magnetic ink sediment							
	Fe	Ti	Cr	Mn	Zn	Si	S	Dry weight (g)
1 M	90.68	I.D.	1.63	I.D.	I.D.	1.36	0.4	0.2018
2 M	95.92	I.D.	I.D.	0.92	0.6	1.24	0.23	0.1857
3 M	99.03	I.D.	I.D.	0.24	I.D.	0.32	0.18	0.0193
4 M	93.03	1.13	I.D.	I.D.	I.D.	5.13	0.35	0.1114
5 M	95.31	1.27	0.17	I.D.	I.D.	I.D.	I.D.	0.0268
6 M^a^	I.D.	I.D.	I.D.	I.D.	I.D.	I.D.	I.D.	–
7 M	95.19	2.19	I.D.	I.D.	I.D.	2.05	0.39	0.1327
8 M	93.27	1.01	I.D.	I.D.	I.D.	3.17	0.25	0.0014
9 M	96.19	0.36	0.12	I.D.	I.D.	1.28	0.36	0.1198
10 M	94.93	1.89	I.D.	0.44	I.D.	1.99	0.78	0.0115

^a^ 6 M nonmagnetic negative control.

I.D., under the detection limit.

Prior to analysis by Mössbauer spectroscopy, two samples were excluded. One sample was lost; another was depleted since too little material was available for further analysis (Figure 1). Final analysis by Mössbauer spectroscopy included in total 8 samples, 7 magnetic and one nonmagnetic (6 M) serving as a negative control.

Mössbauer spectroscopy showed all magnetic samples except sample 1 M, were dominated by the magnetic iron oxide magnetite (Fe_3_O_4_), accompanied by goethite (Fe(OH)O) and sporadic traces of hematite (Fe_2_O_3_) (Table 4). The control sample 6 M, contained no magnetic mineral in agreement with findings by ICP‐MS and XRF showing no iron. Confirmatory analysis using powder X‐ray diffraction supported findings by Mössbauer spectroscopy (Table 4).

**TABLE 4:** Identification of magnetic minerals by Mössbauer spectroscopy and powder X‐ray diffraction (XRD)

	MöSSBAUER SPECTROSCOPY	POWDER X‐RAY DIFFRACTION (XRD)
1 M	N/A, poor quality	Goethite
2 M	Magnetite (56/43), traces of goethite	Magnetite dominated, traces of goethite
3 M	Magnetite (36/52) and goethite	Magnetite and goethite
4 M	Magnetite (42/57)	Magnetite and minor trace of goethite
6 M^a^	N/A	Unknown 3.28 Å
7 M	Magnetite (42/58)	Magnetite and minor trace of goethite
8 M	Magnetite (34/32), goethite, and hematite	Magnetite, goethite, and hematite
9 M	Goethite dominates, magnetite and hematite	Goethite, magnetite, and hematite plus unidentified 9.3Å, 7Å, 14Å

^a^ 6 M nonmagnetic negative control.

N/A, not applicable.

## 4 DISCUSSION

All magnetic ink samples contained metals particularly iron oxides and, as an original finding, the classical iron oxide minerals known from the literature to be magnetic, e.g., magnetite, goethite, and hematite. Magnetic chemicals in the inks are supposed to be outside the recipe and thus not added deliberately; they were seen as impurities originating from the crude pigment raw material.

The concentration of magnetic iron oxide impurities in tattoo inks is not supposed to be linearly correlated to the magnetic property of the product since magnetic property is known in physics to depend on several factors such as oxidation level and crystallographic structure. For instance, both hematite and magnetite are iron oxides but represent different crystal classes with distinct magnetic properties. Hematite is a hexagonal mineral and a spin‐canted antiferromagnet leading to only feeble magnetization whereas magnetite is a hexoctahedral mineral, in an inverse spinel structure leading to a magnetization hundred times stronger. Therefore, these minerals are bound to behave very differently when exposed to MRI conditions. Magnetite is the stronger one. Goethite and hematite are only weakly magnetic in the natural environment. Thus, magnetite is expected to be the lead substance behind the reported MRI adverse reactions of cosmetic tattoo pigments in the skin in situ. Magnetite is the most widely distributed ferrimagnetic, naturally occurring mineral on earth found in igneous and metamorphic rocks. Magnetite has a black‐brown color tone alike the melanin skin pigment, hematite reddish‐brown tone, and goethite yellowish‐brown tone.

Iron oxide pigments have been used in arts and tattooing for thousands of years exemplified by the Lascaux cave painting in France. Today, mineral pigments are widely used in paints, paper, plastic, rubber, and textiles as well as in cosmetic products and medicines as colorants. The benefit of iron oxide pigments is low price and acceptable color robustness on sun exposure and other ambient conditions; and they are well tolerated and cause no allergy. Since the introduction of modern organic tattoo pigments having more intense colors, the use of inorganic pigments with dull colors has declined steeply in use in traditional decorative tattooing but iron oxide pigments with their dull color tones matching facial complexions remain popular in PMU.

Our study identified magnetic impurities in iron oxide‐based tattoo ink products used for permanent makeup. It is likely that magnetite directly causes the instant‐onset and instant‐offset of pain, itch, and burn sensation in the tattoo when MRI is applied, or the patient is moved in the static magnetic fringe field. However, our study cannot explain the precise mechanism of action when an MRI burn is felt by a person. The sensation must involve stimulation of the sensory axon and nerve ending directly in the tattoo. Sensations are mediated to the brain by sensory nerves through propagating depolarization of the axonal membrane potential, thus, by electrical impulse.

In 1820 the Danish physicist Hans Christian Ørsted discovered electro‐magnetism; in the 1830s the British physicist Michael Faraday through elaborate studies showed that an electrical current had a corresponding magnetic field and that changing one could induce its counterpart; the phenomenon of induction. The Inductive force from a strong magnetic field has been applied to clinical medicine to influence neural function directly. E.g. Transcranial Magnetic Stimulation (TMS or rTMS), is mainly used in psychiatry for the treatment of depression, positioned to replace or reduce the use of electroconvulsive therapy (ECT).^17^ TMS is also applied to pain and post‐acute motor stroke supported by higher evidence in the literature.^17^, ^18^, ^19^ Established ECT used routinely for decades itself documents the key role of electrical signaling in neurophysiology.

Tattoo pigment is by electron microscopy of tattooed skin observed directly in the perineurium of cutaneous nerves; closeness between pigment and nerve supports the hypothesis that MRI‐induced magnetic activation of perineural pigment can trigger an electrical signal that makes the sensory nerves lead impulses to the brain, with the sensation being read and felt as burn and pain.^20^ The electron microscopy study includes particle position and physical orientation under the influence of MRI fields; a physical drag effect on pigment particles was so far not observed.^21^

There is in the medical field of tattoo complications an exceptional condition of constant and severe pain in the tattoo contrasting no inflammation and no objective sign of a local reaction, independent of MRI;^22^, ^23^ this condition, in literature named neurosensory reaction, also might be explained by periaxonal pigment influencing the electrical membrane potential of sensory axons supplying the tattoo. It is not known if MRI can trigger a pain attack in this chronic and cumbersome condition. Tattoo and pain or painful sensation resembling burning, thus, are not limited to MRI exposure of the tattoo.

Tattoo pain attack on MRI is, furthermore, not limited to dark cosmetic tattoos, iron oxide impurity, and magnetite; other classes of pigment and ink may exceptionally show a similar sensory response. A recent case of reproducible painful MRI sensation in black tattoos showed no magnetite in the skin by Mössbauer spectroscopy; the culprit pigment was carbon black thus not an iron oxide.^20^ A previous case report of immediate pain sensation in a fresh decorative tattoo already on exposure to the static magnetic field in the surroundings of the MRI scanner in the experimental part of the report also addressed electrical events induced by MRI.^12^ Detailed experimental study on magnetic resonance and electrical conductance and impedance of different classes of pigments indicated a broader range of pigments including organic pigments and carbon black might react electrically on exposure to MRI.^24^ Carbon materials may act as electrical semiconductors. Electrical properties of pigments, determining pigment aggregation ink stock products, are supposed to depend on the electrical potential of the particle surface, measured as the zeta. The zeta potential is influenced by pigment coatings commonly used in ink manufacturing independent of pigment class.^25^ The zeta potential of iron oxides, carbon black, and most other pigments are measured negative. Differences between the potential of particles and the close surrounding create electrical fields, which under the influence of a powerful magnetic field might induce a sensory response.

The immediate onset and end of the MRI burn sensation in hitherto tolerated and noninflamed tattoos, geared by switch on‐and‐off of the scanner, excludes a cell‐mediated response from some pre‐existing lymphocytic infiltrate and its mediators. However, reactive oxygen species (ROS) are instant acting and considered causative in sun sensitivity with phototoxic events as exemplified by congenital erythropoietic protoporphyria; sun immediately induces severe pain, and shadow produces immediate relief. ROS activity easily raises in normal‐appearing skin. There is, however, no indication that persons with tattoos and MRI burn sensation have inherited disease or abnormal reaction pattern in such direction. Iron dextran was shown to stimulate ongoing ROS‐induced skin inflammation but not confirmed itself to initiate ROS reactions.^26^, ^27^ However, ROS and MRI burn sensation occasionally manifested with redness and dermal swelling additional to strong burn sensation should be further studied and the mechanism clarified; neural activity can itself induce inflammation with redness and swelling, known as neurogenic inflammation. ROS activation, however, may easily explain increased sun sensitivity of tattoos with pain and burn sensation, the commonest tattoo complaint noted mostly in the dark‐colored decorative tattoos.^28^

MRI‐induced sensations and reactions in tattoos may become more common in the future when the younger generations after some years may need MRI evaluation of some suspected cancer or other age‐related diseases. Tattoos are long lasting and MRI complications may occur with decades of delay as already noted in the literature. The problem of MRI burn sensation in tattoos is according to a Swedish study from MRI units heavily underreported.^29^

Our observation that iron oxide‐based ink stock products commonly are magnetic contrasts no indication that every person tattooed with magnetic ink will risk MRI burn sensation. Present clinical evidence indicates only 1–2% of tattooed patients react in their tattoo on MRI.^6^ Obviously, strong individual and local prerequisites or factors diminish the response rate; it may be the density and closeness of pigment in situ nearby the axon; it may be variable axonal threshold for this kind of sensation; and it may be the coating of pigment particles to influence their electrical surface potential and the electrical field relative to the pigment core. There may also be a window of increased sensitivity to MRI in the fresh tattoo in the healing phase before washout of tattoo pigment and metals seeded by tattoo needling is effective.^30^

## 5 CONCLUSION

We report the original observation that iron oxide pigments used in cosmetic tattoo ink stock products commonly contain magnetic iron oxide impurities namely magnetite, goethite, and hematite. Magnetite is considered the main component responsible for instant burn sensations in cosmetic tattoos exposed to MRI. A simple magnet test of ink stock products with a handheld magnet can be used to distinguish magnetic and nonmagnetic inks. Used for product screening as a safety measure, the test is limited by many false‐positive outcomes versus clinical adverse response. A range of individual and local factors and other ink‐related conditions appear involved. It is hypothesized that the mechanism of MRI tattoo reactions is a magnet‐electrical induction of pigment particles nearby neurons resulting in direct excitation of sensory neurons in the tattoo with a sensory signal read by the central nervous system as a painful burn sensation in the tattoo.

## Data Availability

The data that support the findings of this study are available from the corresponding author upon reasonable request.
